# Systematic relationships of five newly sequenced cervid species

**DOI:** 10.7717/peerj.2307

**Published:** 2016-08-04

**Authors:** Nicola S. Heckeberg, Dirk Erpenbeck, Gert Wörheide, Gertrud E. Rössner

**Affiliations:** 1 Department for Earth and Environmental Sciences, Palaeontology & Geobiology, Ludwig-Maximilians-Universität München, Munich, Germany; 2 SNSB-Bayerische Staatssammlung für Paläontologie und Geologie, Munich, Germany; 3 Department of Zoology, University of Cambridge, Cambridge, United Kingdom; 4 GeoBio-Center, Ludwig-Maximilians-Universität München, Munich, Germany

**Keywords:** Cervidae, Phylogeny, Polyphyly, Cytochrome b, *Pudu*, *Mazama*, *Muntiacus*, *Rusa*

## Abstract

Cervid phylogenetics has been puzzling researchers for over 150 years. In recent decades, molecular systematics has provided new input for both the support and revision of the previous results from comparative anatomy but has led to only partial consensus. Despite all of the efforts to reach taxon-wide species sampling over the last two decades, a number of cervid species still lack molecular data because they are difficult to access in the wild. By extracting ancient DNA from museum specimens, in this study, we obtained partial mitochondrial cytochrome b gene sequences for *Mazama bricenii*, *Mazama chunyi*, *Muntiacus atherodes*, *Pudu mephistophiles*, and* Rusa marianna*, including three holotypes. These new sequences were used to enrich the existing mitochondrial DNA alignments and yielded the most taxonomically complete data set for cervids to date. Phylogenetic analyses provide new insights into the evolutionary history of these five species. However, systematic uncertainties within *Muntiacus* persist and resolving phylogenetic relationships within *Pudu* and *Mazama* remain challenging.

## Introduction

Cervidae forms a subclade of ruminant artiodactyls and is the second most diverse group among terrestrial artiodactyls, with 55 extant species (IUCN, 2015), including one recently extinct species (*Rucervus schomburgki*; Duckworth, Robichaud & Timmins, 2008). Cervids natively inhabit Eurasia, the Americas, and potentially northernmost Africa (Mattioli, 2011). They are adapted to diverse climatic zones, ranging from the tropics to arctic regions, and to diverse habitats such as tundra, grasslands, swamps, forests, woodlands, and ecotones (Mattioli, 2011). Their unique phenotypic feature is a pair of antlers, which are osseous outgrowths of the frontal bone that are shed and rebuilt regularly. The current conservation status of cervids lists 29 species as ‘threatened’, nine species as ‘data deficient’, and 17 species as ‘least concern’ in the IUCN Red List of Threatened Species (IUCN, 2015). Samples and life history data are much more difficult to obtain from rare and threatened species than from more abundant species. Therefore, there is a discrepancy between the well-studied (e.g., *Cervus elaphus*, red deer; *Odocoileus hemionus*, mule deer; *Rangifer tarandus*, reindeer) and barely known species (e.g., *Mazama* spp., brocket deer; *Pudu* spp., pudu; *Muntiacus* spp., muntjac). Consequently, data for the latter taxa are overdue.

Cervid phylogenetics has improved considerably in recent decades through molecular systematics (e.g., Hassanin & Douzery, 2003; Pitra et al., 2004; Kuznetsova, Kholodova & Danilkin, 2005; Hernández-Fernández & Vrba, 2005; Hughes et al., 2006; Gilbert, Ropiquet & Hassanin, 2006; Marcot, 2007; Agnarsson & May-Collado, 2008; Duarte, González & Maldonado, 2008; Hassanin et al., 2012). However, several species are still underrepresented in molecular phylogenetic analyses because their current conservation status of threatened or data deficient negatively affects their sample collection.

**Figure 1:**
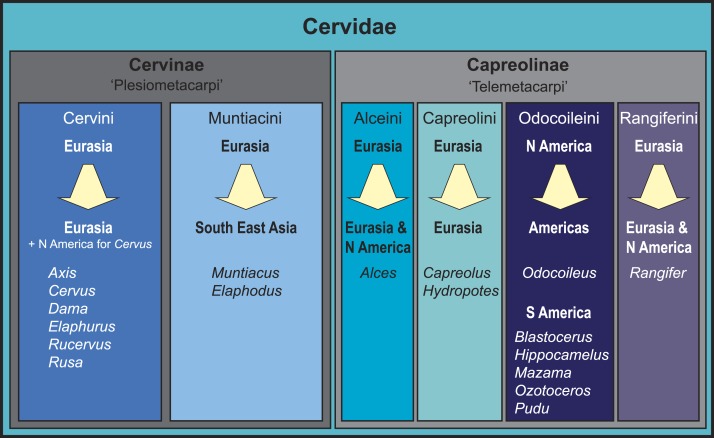
Overview of the current state of the art of cervid classification based on literature (e.g., Hassanin & Douzery, 2003; Pitra et al., 2004; Kuznetsova, Kholodova & Danilkin, 2005; Hernández-Fernández & Vrba, 2005; Hughes et al., 2006; Gilbert, Ropiquet & Hassanin, 2006; Marcot, 2007; Agnarsson & May-Collado, 2008; Duarte, González & Maldonado, 2008; Hassanin et al., 2012). The diagram shows the different clades, their geographical origination, and their current distribution.

Consensus has been reached for the monophyly of taxa Cervidae, Muntiacini, Cervini, Capreolini and Odocoileini. Muntiacini and Cervini form the clade Cervinae, which is a sister taxon to Capreolinae comprising Odocoileini, Rangiferini, Capreolini and Alceini (e.g., Hernández-Fernández & Vrba, 2005; Gilbert, Ropiquet & Hassanin, 2006; Hassanin et al., 2012). The Capreolinae-Cervinae-split is commonly supported in previously published topologies and corresponds to the first (though not formally valid) morphological cervid classification by Brooke (1878), who differentiated Plesiometacarpi and Telemetacarpi (Fig. 1). Systematic relationships within Cervinae appear to be largely resolved, whereas Capreolinae systematics is more controversial (Pitra et al., 2004; Gilbert, Ropiquet & Hassanin, 2006; Duarte, González & Maldonado, 2008; Hassanin et al., 2012; Croitor, 2014). For an overview of current cervid classifications, see Fig. 1.

The mitochondrial cytochrome b (*Cytb*) gene is the best-sampled across cervids. *Cytb* is a marker that is known to be highly variable in mammals, which makes it a suitable marker for resolving genus and species level relationships but less suitable for resolving deeper nodes (family level and above) or for population studies (Hofreiter et al., 2001a). In addition, because mitochondrial genomes are maternally inherited, they may not allow a full reconstruction of a species’ evolutionary history if there is no random mating.

However, Hassanin et al. (2012) sequenced and analysed mitochondrial genomes of 33 cervid species as part of a large Artiodactyla phylogenetic reconstruction and provided a robust phylogenetic framework for cervids. To date, sampling of mitochondrial genomes and individual partial *Cytb* sequences cover 46 of the 55 cervid species.

Here, we present the results of phylogenetic analyses that include four species not previously sampled for molecular data: *Mazama chunyi* (Peruvian dwarf brocket), *Muntiacus atherodes* (Bornean yellow muntjac; including holotype), *Pudu mephistophiles* (Northern pudu; including holotype), and *Rusa marianna* (Philippine brown deer), all of which were taken from museum specimens. We also sequenced three *Mazama bricenii* museum specimens (Mérida brocket; including the holotype), of which *Cytb* sequences have been published recently and were sequenced contemporaneously with our study (Gutiérrez et al., 2015). Except for *M. atherodes* (least concern), all species have been assessed as vulnerable based on the IUCN Red List. Therefore, considering the threat of extinction, our approach of sequencing DNA from museum material is an important contribution to cervid systematics.

The specific aims of our study were (1) to reconstruct the systematic position of *M. bricenii* and *M. chunyi* and further investigate the polyphyly of the genus *Mazama*, (2) to reconstruct the systematic position of *M. atherodes*, (3) to test the monophyly of the Philippine *Rusa* species (*R. alfredi* and *R. marianna*) and their sister taxon position relative to the Indonesian and mainland *Rusa* species (*R. timorensis* and *R. unicolor*), and (4) to test the monophyly of *Pudu*.

To achieve these aims, we experimented with different matrix sizes and parameters to examine the reliability of the phylogenetic signal throughout different data sets.

## Material & Methods

### Material

**Figure 2:**
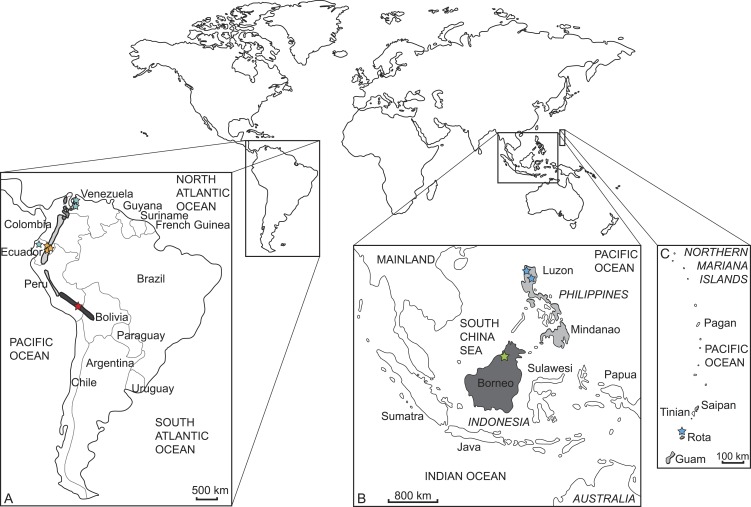
Current distribution of the sampled species and the approximate sampling localitions of the specimens. (A) Enlarged map of South America; dark grey/red star: *Mazama chunyi*, light grey/yellow stars: *Pudu mephistophiles*, medium grey/turquoise stars: *Mazama bricenii*. (B) Enlarged map of Indonesian and Philippine Islands and (C) enlarged map of the Northern Mariana Islands; dark grey/green star: *Muntiacus atherodes*, light grey/blue stars: *Rusa marianna*.

We sampled and sequenced five species from which no molecular data were available previous to our study (but see Gutiérrez et al., 2015) (Tables 1 and 2). Samples were taken from thirteen museum specimens, nine from the Natural History Museum in London (BMNH) and four from the Museum für Naturkunde Berlin (ZMB). Three specimens represent holotypes (BMNH 1908.6.24.5 *Mazama bricenii*, BMNH 1971.3088 *Muntiacus atherodes*, BMNH 1896.1.28.6 *Pudu mephistophiles*). One sample was derived from a wet specimen, one from a skin, and the remaining samples consisted of bone fragments or dried soft tissue remains of skulls (details in Table 2). Figure 2 shows where the specimens originated and their currently known species distributions. The collection dates of each specimen are given in Table 2.

**Table 1:** GenBank and ENA accession numbers. Newly sequenced species are in bold.

Species	Cytochrome b	mtGenome
*Alces alces*	AJ000026	JN632595
*Alces americanus*	M98484	–
*Axis axis*	AY607040	JN632599
*Axis kuhlii*	HQ893538	–
*Axis porcinus*	DQ379301	JN632600
*Blastocerus dichotomus*	JN632603	JN632603
	AY607038	
	NC_020682	
*Capreolus capreolus*	AJ000024	JN632610
*Capreolus pygargus*	AJ000025	–
*Cervus albirostris*	AY044863	JN632690
	AF423202	
*Cervus elaphus canadensis*	AF423198	–
	EF139147	
*Cervus elaphus*	JF489133	NC_007704
*Cervus nippon*	JF893484	NC_006993
*Dama dama*	AJ000022	JN632629
*Dama mesopotamica*	AY607034	JN632630
*Elaphodus cephalophus*	NC_008749	NC_008749
*Elaphurus davidianus*	AF423194	JN632632
*Hippocamelus antisensis* 1	JN632646	JN632646
	NC_020711	
*Hippocamelus antisensis* 2	DQ379307	
	GU190862	
*Hippocamelus bisulcus*	DQ789177	–
	DQ789178	
	GU190863	
*Hydropotes inermis*	AJ000028	JN632649
*Mazama americana* 1	DQ789209	JN632656
	DQ789217	
*Mazama americana* 2	JN632657	
*Mazama americana* 3	DQ789221	
	JN632656	
	NC_020719	
*Mazama americana* 4	DQ789201	
	DQ789204	
*Mazama americana* 5	DQ789219	
*Mazama bororo*	DQ789187	–
	DQ789231	
	DQ789228	
***Mazama bricenii***	**LT546656**	**–**
	**LT546657**	
	**LT546658**	
***Mazama chunyi***	**LT546655**	**–**
*Mazama gouazoubira* 1	JN632658	JN632658
	NC_020720	
*Mazama gouazoubira* 2	DQ379308	
*Mazama nana*	DQ789210	–
	DQ789214	
	DQ789227	
*Mazama nemorivaga* 1	JN632660	JN632660
*Mazama nemorivaga* 2	DQ789205	
	DQ789206	
	DQ789226	
	JN632659	
	NC_024812	
*Mazama pandora*	KC146954	–
	KC146955	
*Mazama rufina*	JN632661	JN632661
	NC_020721	
*Mazama temama*	KC146956	–
	KC146957	
	KC146958	
	KC146959	
† *Megaloceros giganteus*	AM182644	–
	AM182645	
***Muntiacus atherodes***	**LT546659**	**–**
*Muntiacus crinifrons*	NC_004577	NC_004577
	AY239042	
	DQ445734	
	DQ445732	
	DQ445735	
	DQ445733	
*Muntiacus feae*	AF042721	–
*Muntiacus muntjak* 1	NC_004563	NC_004563
	AY225986	
*Muntiacus muntjak* 2	AF042718	
*Muntiacus putaoensis*	EF523665	–
	EF523666	
	EF523667	
	EF523668	
	EF523669	
*Muntiacus reevesi*	AF527537	NC_008491
	NC_004069	
*Muntiacus rooseveltorum*	KJ425278	–
	KJ425279	
	KJ425281	
	KJ425282	
*Muntiacus truongsonensis* 1	KJ425277	–
*Muntiacus truongsonensis* 2	KJ425276	
*Muntiacus vuquangensis*	FJ705435	FJ705435
	AF042720	
	NC_016920	
*Odocoileus hemionus* 1	HM222707	JN632670
*Odocoileus hemionus* 2	FJ188783	
	FJ188870	
*Odocoileus virginianus* 1	DQ379370	JN632671
*Odocoileus virginianus* 2	M98491	
*Ozotoceros bezoarticus*	DQ789190	JN632681
	DQ789193	
	DQ789195	
	DQ789199	
*Pudu mephistophiles*	JN632691	–
	**LT546651**	
	**LT546652**	
	**LT546653**	
	**LT546654**	
*Pudu puda*	JN632692	JN632692
	AY607039	
	NC_020740	
*Rangifer tarandus*	AB245426	NC_007703
	AY726704	
	KM506758	
*Rucervus duvaucelii*	AY607041	JN632696
*Rucervus eldii*	AY157735	JN632697
*Rucervus schomburgki*	AY607036	–
*Rusa alfredi*	JN632698	JN632698
	NC_020744	
***Rusa marianna***	**LT546647**	**–**
	**LT546648**	
	**LT546649**	
	**LT546650**	
*Rusa timorensis*	AF423200	JN632699
*Rusa unicolor*	FJ556575	NC_008414
*Antilocapra americana*	JN632597	JN632597
*Boselaphus tragocamelus*	EF536350	EF536350
*Hyemoschus aquaticus*	JN632650	JN632650
*Moschus moschiferus*	FJ469675	JN632662
*Okapia johnstoni*	JN632674	JN632674
*Tragelaphus scriptus*	AF022067	JN632706

We obtained complete *Cytb* and/or mitochondrial genome sequences from NCBI GenBank (http://www.ncbi.nlm.nih.gov/genbank/) for 48 cervid species. These included the 45 extant cervids (full set of available extant cervid data excluding recently published *M. bricenii* sequences; Gutiérrez et al., 2015), one subspecies (*Cervus elaphus canadensis*), a questionable *P. mephistophiles* sequence from Hassanin et al. (2012), and one fossil cervid species (*Megaloceros giganteus*). We also added six non-cervid ruminant taxa (Table 1). The resulting *Cytb* data set is the most taxonomically extensive for Cervidae to date.

**Table 2:** Overview of sampled specimens. Specimens in bold are holotypes. The category ‘sample DNA’ provides the weight of the tissue sample in the tube prior to DNA extraction and DNA concentration after extraction.

Species	Collection ID	Accession no.	Sample (mg)	DNA (ng/µl)	Gaps in alignment	Collection entry	Locality	Material
*Rusa marianna*	BMNH 1996.2	LT546647	15.5	93.65	–	1996	Philippines	Soft tissue fragments^*^
*Rusa marianna*	ZMB-MAM-75158	LT546648	15.1	60.97	–	NA	Philippines, Luzon	Soft tissue & bone fragments^*^
*Rusa marianna*	ZMB-MAM-20409	LT546649	12.0	49.64	–	1915	Captive animal	Soft tissue & bone fragments^*^
*Rusa marianna*	ZMB-MAM-75146	LT546650	26.2	38.67	403–467	1905	US, Northern Mariana Islands	Soft tissue & bone fragments^#^
*Pudu mephistophiles*	BMNH 1899.2.18.20	LT546651	30.5	97.35	64–118, 176–211	1899	Ecuador	Soft tissue & bone fragments^*^; juvenile
***Pudu mephistophiles***	BMNH 1896.1.28.6	LT546652	7.6	56.57	–	1896	Ecuador, Paramo of Papallacta	Snippet of skin, including hair; immature
*Pudu mephistophiles*	BMNH 1899.2.18.21	LT546653	9.9	27.34	604–674, 784–810	1899	Ecuador	Soft tissue & bone fragments^*^; juvenile
*Pudu mephistophiles*	ZMB-MAM-61577	LT546654	165.8	325.57	–	1970	Captive animal	Wet specimen; neonatal
*Mazama chunyi*	BMNH 1967.1362	LT546655	15.6	56.22	–	1967	Peru, Chiquis	Soft tissue & bone fragments^**^
*Mazama bricenii*	BMNH 1913.4.24.3	LT546656	36.0	74.17	–	1913	Venezuela, Merida	Soft tissue & bone fragments^*^
***Mazama bricenii***	BMNH 1908.6.24.5	LT546657	2.4	7.07	288–394, 604–674	1908	Venezuela	Soft tissue & bone fragments^*^
*Mazama bricenii*	BMNH 1934.9.10.228	LT546658	10.2	77.08	–	1934	Ecuador, Pichincha	Soft tissue & bone fragments^*^
***Muntiacus atherodes***	BMNH 1971.3088	LT546659	23.3	87.60	–	1971	Borneo, Brunei/ Indonesia/Malaysia	Soft tissue & bone fragments^**^

**Notes.**

TITLE BMNH British Museum of Natural History London ZMB Zoological collections of the Museum für Naturkunde Berlin

* From skull.

** From skull & mandible.

# From mandible.

### Extraction

The challenges of sequencing ancient DNA are related to the degradation of DNA after an organism’s death triggered by exogenous processes such as oxidation and background radiation. These processes affect the sugar-phosphate backbone and nitrous bases of the DNA strand, whereas hydrolytic processes such as depurination and deamination cause breakage in the DNA molecules (Hofreiter et al., 2001b). Due to the large number of mitochondria per cell, mitochondrial gene sequences are more likely to be retrieved from ancient material than is nuclear DNA (Hofreiter et al., 2001a).

DNA was extracted using the Qiagen QIAamp DNA Micro Kit, including an overnight lysis step, following the manufacturer’s protocol. After lysis, 1 µg dissolved carrier RNA was added, as recommended in the protocol, 80 µl elution buffer was used for the last elusion step, and the last incubation step was set for five minutes instead of one minute. After the extraction, the DNA concentration was measured using a spectrometer (NanoDrop 1000; Peqlab Biotechnologie GmbH, software version ND 1000 v3.7.1) (Table 2).

### PCR

Eight cervid-specific *Cytb* primers (Lister et al., 2005) were used to amplify a 747 base pair region from the 1140-base-pair-long mitochondrial *Cytb*, from nucleotide position 64 to 810. Each primer pair amplified a 100–140-base-pair-long sequence with overlap to adjacent sequences (Lister et al., 2005; Table 2).

**Table 3:** PCR recipes. Initial PCRs were undertaken using recipe (a), for optimisation recipes (b)–(d) were used depending on fragment and sample. Reagents that were varied are in bold. Components of column (a) in combination with an annealing temperature of 50 °C worked better for primer pair 4, (d) worked well for primer pair 8, and (c) worked better for some samples in combination with primer pair 2 (Lister et al., 2005). Except for one case, varying the annealing temperature had no influence on the reaction.

**Reagents**	**Quantity (µl)**			
	a	b	c	d
PCR Flexi-Buffer (5X)	2.5	2.5	2.5	5
MgCl_2_ (25 mM)	**1.5**	**1.5**	**2**	**3**
dNTPs (10 mM)	0.5	0.5	0.5	1
Primer forward (5 µM)	0.5	0.5	0.5	1
Primer reverse (5 µM)	0.5	0.5	0.5	1
BSA	**1.3**	**0**	**0**	**0**
H_2_O	4.6	5.9	5.4	12.9
Go*Taq* polymerase	0.1	0.1	0.1	0.1
DNA	1	1	1	1
**Total reaction volume**	**12.5**	**12.5**	**12.5**	**25**

Polymerase chain reactions (PCR) were carried out using a TProfessional thermocycler (Biometra). Sequences amplified from each primer pair were validated against contamination with a negative control. The specific PCR components are given in Table 3. The PCR programme was as follows: initial denaturation at 95 °C for three minutes, then 35 cycles of denaturation at 95 °C for 30 s, annealing at 55 °C for 30 s, and extension at 72 °C for 30 s, and a final extension at 72 °C for five minutes. Amplification of target sequences was initially attempted using the components in Table 3, column (a) and an annealing temperature of 55 °C. Some primer-sample combinations did not result in amplification products. Therefore, we experimented with the components, e.g., not adding Bovine Serum Albumin (BSA), changing the overall reaction volume, and/or increasing the concentration of magnesium chloride (Table 3). We also experimented with annealing temperatures ranging from 48 °C to 52 °C. These optimisations were successful in most cases; however, a few sections of the individual sequences for certain specimens could not be successfully amplified, which left gaps in the *Cytb* sequence (Table 2).

Successfully amplified PCR products were sequenced in both directions using the amplification primers and the ABI BigDyeTerminator 3.1 chemistry following the manufacturer’s protocol on a capillary sequencer (ABI 3730; AppliedBiosystems) in the Genomic Sequencing Unit, Faculty of Biology, LMU. After quality control, the approximately 100–140-base-pair-long forward and reverse sequencing reads were assembled into contigs. These individual contigs were then assembled into a contig with a maximum length of 747 base pairs using CodonCodeAligner v.3.7.1.1.

To ensure that a genuine cervid *Cytb* fragment has been amplified, the forward and reverse pre-assembly sequences from each primer, the individual contigs of forward and reverse strands and the final 747-base-pair-long contigs were each BLASTed against NCBI GenBank entries. Only fragments returning a cervid in the first 50 BLAST search results were used. In almost all cases, where the BLAST result was different from the cervid result, the sequences were found to be most similar to *Bos taurus*. This contamination is possibly caused by the BSA added to enhance PCR outcomes. Sequences were submitted to the European Nucleotide Archive under accession numbers LT546647 –LT546659 (Tables 1 and 2).

### Alignment

The concatenated consensus sequences of each specimen were added to the existing *Cytb* data set (NCBI GenBank) and pairwise aligned by eye using Mesquite v.2.75 (Maddison & Maddison, 2011) and Seaview 4.2 (Gouy, Guindon & Gascuel, 2010). The alignment was carefully checked for stop codons within the alignment and/or unusual nucleotide positions by translation into amino acids to ensure the absence of pseudogenes and sequencing errors. The IUPAC ambiguity code was used in few cases where character states could not be assessed unambiguously after a re-investigation of the raw sequence data. These ambiguities most likely represent misreads from the chromatogram due to the somewhat poor condition of the DNA. Because these ambiguous sites are not numerous, their impact on the phylogenetic signal is negligible.

In total, three different alignments were created. First, we aligned the new 747 base pair long sequences with the complete *Cytb* sequences from GenBank to form a data set of 1140 base pairs. The final data set contained 130 taxa (124 cervids, six other ruminants). Second, to test whether the newly sequenced, shorter fragments carried a sufficient phylogenetic signal, two further alignments were created. One alignment was exactly 747 base pairs long, which was the same length as the new sequences, including internal gaps. The other alignment excluded even the internal gaps and was 569 base pairs long. We also re-analysed the cervid subset (33 species) of the complete mitochondrial genome alignment available for Artiodactyla in Hassanin et al. (2012) without the new sequences. The taxon sampling contained 39 cervid taxa and seven non-cervid ruminants.

### Phylogenetic analyses

To test for the impact of alignment length on phylogenetic signal, we developed three alignments with varying base pair lengths. For each alignment, we used PartitionFinder (Lanfear et al., 2012) to identify the optimal partitioning scheme and mutation model (Table 4).

**Table 4:** Summary of analyses, model choice, partitioning, and support for major clades in the resulting topologies.

Analysis	Reference	Model(s)	Partitioned	Cervidae	Cervinae	Cervini	Muntiacini	Capreolinae	Capreolini	Odocoileini	Blastocerina	Odocoileina
BI-mtG	Fig. 3A, Fig. S1	GTR	Y	1	1	1	1	1	1	1	1	1
BI-1140-unpart	Fig. 3B, Fig. 4, Fig. S2	GTR	N	1	1	1	1	–	1	.84	.98	.99
BI-1140-part	Fig. 3C, Fig. S3	SYM	Y	1	.99	1	1	–	1	–	.75	.87
		HKY										
		GTR										
ML-1140	Fig. 3D, Fig. S4	GTR	N	99	89	99	92	–	100	57	55	41
BI-747-unpart	Fig. 3E, Fig. S5	GTR	N	1	1	1	.81	–	1	–	.85	–
BI-747-part	Fig. 3F, Fig. S6	SYM	Y	1	.99	1	.90	–	1	–	–	–
		HKY										
		GTR										
BI-569-unpart	Fig. 3G, Fig. S7	GTR	N	1	–	.99	.92	–	1	–	–	–

**Notes.**

Abbreviations: BI Bayesian Inference ML Maximum Likelihood, the number represents the *Cytb* sequence length in the current alignment Y yes N no part partitioned unpart unpartitioned

The values within cells represent the node support for the respective split either as Bayesian posterior probabilities or as bootstrap support from maximum likelihood analyses; “–” indicates that the clade was not recovered in the respective analysis.

A summary of all analyses undertaken including the models and partitioning scheme, is shown in Table 4. PartitionFinder analysis on the 1140 *Cytb* data set resulted in a scheme with three different partitions for the individual codon positions using SYM for position 1, HKY for position 2, and GTR for position 3 for Bayesian inference analyses with MrBayes v.3.2.4 (Ronquist et al., 2012) (in the following referred to as BI-1140-part). For the maximum likelihood analyses with RAxML (Stamatakis, 2006), PartitionFinder suggested GTR for all codon positions (ML-1140). Alternatively, we undertook a Bayesian inference analysis without partitioning using the GTR model on the 1140-base-pair-long alignment (BI-1140-unpart). We also undertook a Bayesian analysis with the *Cytb* alignment reduced to 747 base pairs (BI-747-part) using the partitioning scheme suggested by PartitionFinder and the models decribed above as well as one unpartitioned analysis (BI-747-unpart) using GTR. Further, we undertook another Bayesian analysis on the 569 base pair alignment (BI-569-unpart), excluding the internal gaps, representing the shortest sequence length of the newly sequenced taxa (Maz_bri_Q_BMNH_1908.6.24.5). This analysis was run using the GTR model and no partitioning because of the short alignment length. The Bayesian re-analysis of the complete mitochondrial genome sequences (BI-mtG; without the newly sequenced *Cytb* sequences) was undertaken using GTR and divided the data set into seven partitions (Hassanin et al., 2012). The re-analysis was carried out because previous re-analyses of subsets of the complete mitochondrial genome resulted in different results than those found by Hassanin et al. (2012).

Substitution models for all analyses were implemented with a gamma distribution (*Γ*) without a proportion of invariant sites (*I*), although PartitionFinder suggested using Γ + *I* for most partitions. It is known that the combination Γ + *I* may create two areas of equal probability in the tree landscape, which can lead to convergence problems (Moyle et al., 2012). All Bayesian Inference analyses were run with MrBayes v.3.2.4 (Ronquist et al., 2012) using Metropolis-Coupled Markov Chain Monte Carlo (MC^3^); two separate runs sampled the tree landscape at a temperature of 0.35 sampling every 1,000th tree. The mitochondrial genome analysis was run with MrBayes v.3.2.4 (Ronquist et al., 2012) using MC^3^ with two separate runs sampling every 5,000th tree at a temperature of 0.35. All analyses automatically stopped when the standard deviation of split frequencies of posterior probabilities reached 0.01. From all post burn-in sampled trees, a consensus tree was generated (burn-in = 25%). For the Maximum Likelihood analysis we used RAxML v.7.3.0 (Stamatakis, 2006) including a rapid bootstrap search with 100 replicates on the 1140 base pair long data set.

*Hyemoschus aquaticus* (Tragulidae, Artiodactyla), which is an extant representative of crown ruminants, was used as the outgroup. The original tree topologies from all seven analyses are provided in Figs. S1–S7, and an overview is given in Fig. 3 and Table 4.

**Figure 3:**
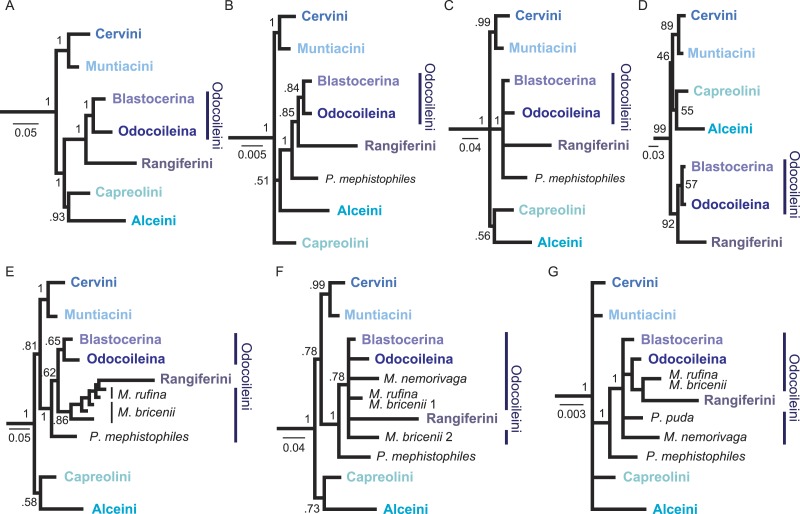
Overview of higher level topologies resulting from re-analysis of the complete mitochondrial genome sequences (Hassanin et al., 2012) and six different analyses of our data set. (A) BI-mtG, (B) BI-1140-unpart, (C) BI-1140-part (D) ML-1140, (E) BI-747-unpart, (F) BI-747-part, (G) BI-569-unpart. Support values represent bootstrap values in D, all other support values are posterior probabilities. (A–D) show monophyly for all major cervid lineages, whereas in (E–G) resolution, particularly within Odocoileini is lost. Positioning *P. mephistophiles* proves to be difficult. Scale bars represent substitutions per site.

**Figure 4:**
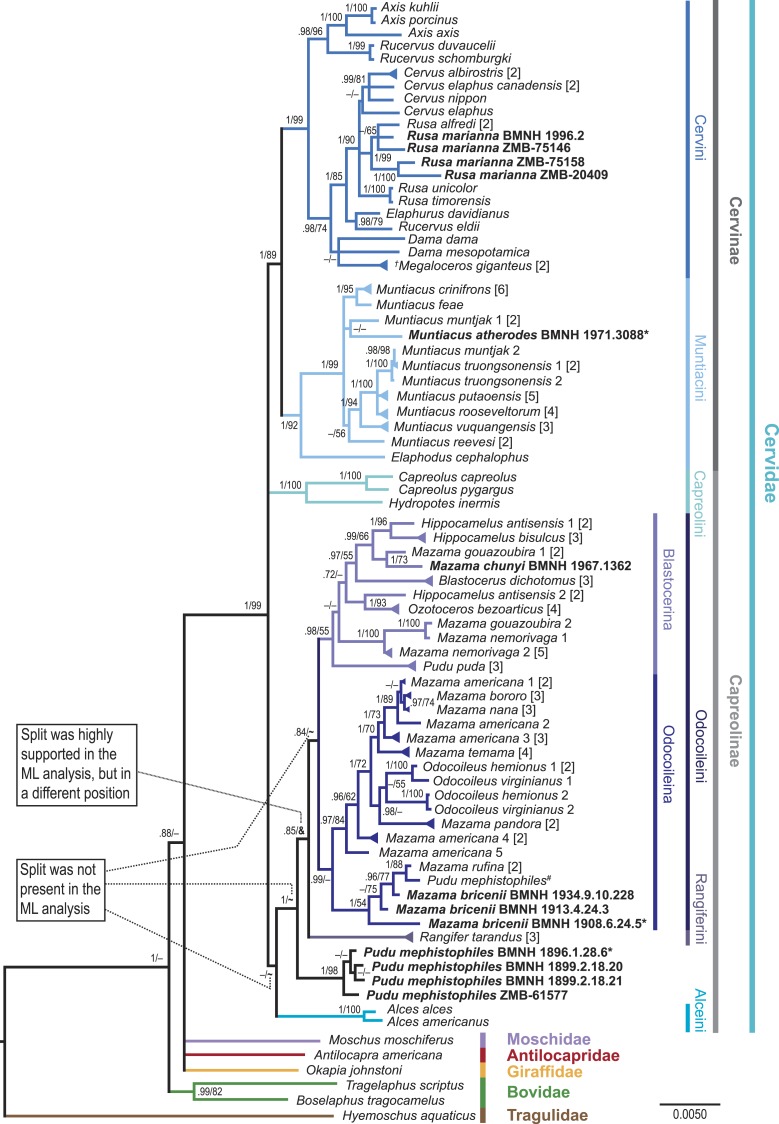
Consensus tree of the unpartitioned Bayesian Analyses (BI-1140-unpart). Values represent posterior probabilities (PP), and if applicable, bootstrap (BS) support from the ML analysis is shown. Only values larger than 70% (PP) and 50% (BS) are displayed. If the support was not above 70% or 50%, but the split was present in one of the analyses; this is indicated by an “–”. “**∼**” indicates that the split was absent in the maximum likelihood analysis. “**&**” indicates that the split was absent in the maximum likelihood topology, but highly supported in a different position; this is only represented in the node separating *Rangifer* from the majority of Odocoileini (see Fig. S4). The numbers in square brackets indicate the number of individual sequences representing the taxon in the present analysis. If these multiple sequences representing one species were not identical, it is indicated by a triangular shaped tip of the branch. Taxa in bold are the newly sequenced specimens, asterisks indicate holotypes, and the hash indicates the putatively wrongly assigned *P. mephistophiles* sequence. Higher hierarchical taxa are shown on the right.

## Results

### Extraction, PCR, sequencing

The results from the DNA extraction, PCR, and sequencing processes are summarised in Table 2. For some of the eight *Cytb* fragments, DNA amplification was not sufficient, which resulted in gaps in the sequence for a few specimens (Table 2). Upon checking the traces in CodonCodeAligner, we observed in our alignment that Y (C or T; *n* = 50) and R (G or A; *n* = 19) are the most common ambiguities. These nucleotide substitutions are most likely caused by hydrolytic deamination. This is a process by which the deamination of cytosine residues to form uracil residues, 5-methyl-cytosine residues to form thymine residues, or adenine residues to form hypoxanthine residues in the template DNA strand will be misread during the PCR process when a new DNA strand is synthesised. In turn, this leads to evident C → T or G → A substitutions (Hofreiter et al., 2001a.; Pääbo et al., 2004; Briggs et al., 2007; Briggs et al., 2010). Across our samples, Y ambiguities occurred up to ten times per specimen, and R ambiguities occurred up to three times per specimen. These numbers represent a very small proportion of approximately 1% of the overall sequence length of 747 base pairs. We tested the impact of the ambiguities on the reconstruction and found that the ambiguities did not tremendously influence the phylogenetic signal of the samples. However, these ambiguities represent an additional uncertainty in the analyses.

### Phylogenetic analyses

The results from seven analyses are summarised in Table 4 and Fig. 3. Of the full 1140-base-pair-long *Cytb* data set 593 characters are constant, 68 variable characters are parsimony-uninformative, and 479 characters are parsimony-informative. The analyses of the 1140-base-pair-long *Cytb* represent our primary results and are shown in Fig. 4. In addition to the Bayesian Inference analyses and the Maximum Likelihood analysis of the total *Cytb* data set (including the new sequences), we tested the impact of reduced data sets (569 characters and 747 characters, Bayesian Inference) and different partitioning schemes on the phylogenetic signal (BI-1140-unpart, BI-1140-part, ML-1140, BI-569-unpart, BI-747-unpart, BI-747-part; Table 4, Fig. 3, Figs. S2–S7).

We next re-analysed the complete mitochondrial genome alignment from Hassanin et al. (2012) for the subset of cervids (14904 base pairs, Bayesian Inference; BI-mtG, Fig. S1), because the authors stated that some of the nodes are not robust, as proven by previous re-analyses (Bibi, 2014). The re-analysis presented here (BI-mtG, Fig. S1) resulted in the support of a fully resolved topology, which is congruent with the topology in Hassanin et al. (2012).

Data partitioning of the 1140-base-pair-long *Cytb* data set and reduced data sets did not lead to contradictory results compared to unpartitioned analyses or larger data sets. Resolution and node supports generally decreased with decreasing alignment length (Fig. 3). Cervid lineages above the genus level were almost always recovered with all matrix sizes and partitioning schemes (Table 4). None of the topologies supportably contradicted each other; however, all topologies differed from each other to some extent at the tribe, genus, and/or species level. Compared to the *Cytb*-only topologies, the mitochondrial genome topology showed generally higher posterior probabilities (Figs. 3 and 4).

The monophyly of superordinate clades, Cervidae, Cervini, Muntiacini, and Capreolini (including* Hydropotes*), was supported in all topologies (Figs. 3 and 4, Figs. S1–S7, Table 4). In all but one topology (BI-569-unpart; Fig. 3G,  Fig. S7), the monophyly of Cervinae, was consistently supported (Fig. 3, Table 4). Odocoileini was weakly supported in three topologies (ML-1140, BI-1140-unpart, BI-mtG; Figs. 3A, 3B , 3D and 4, Figs. S1, S2, S4 and Table 4). Capreolinae, however, was supported as a monophyly in only one topology (BI-mtG, Fig. 3A, Fig. S1), and in the other topologies, the taxon splits unresolved into Odocoileini, Rangiferini (*Rangifer*), Alceini (*Alces*), and Capreolini (*Capreolus*,* Hydropotes*) (Fig. 3). Alceini and Capreolini sometimes formed a clade (Figs. 3A, 3C, 3D, 3E and 3F) or were unresolved (Figs. 3B and 3G). Systematic relationships of capreoline taxa showed marginal differences in each of our topologies.

The results at the genus and species levels are shown in Fig. 4 and Figs. S1–S7. The newly sequenced *Muntiacus atherodes* nested within Muntiacini, mostly polytomous, with two *Muntiacus*-clades. One clade consisted of *M. muntjak, M. feae*, and *M. crinifrons*, and the other consisted of *M. truongsonensis*, *M. putaoensis*, *M. rooseveltorum*, *M. reevesi*, and *M. vuquangensis*. Two topologies (BI-1140-unpart, ML-1140) indicated a poorly supported sister taxon relationship between *M. muntjak* and *M. atherodes* (Fig. 4, Figs. S2 and S4).

We found strong support in Cervini to place all four *Rusa marianna* specimens in a Philippine *Rusa*-clade, with *Rusa alfredi* in all but one topology (BI-569-unpart; Fig. 4, Figs. S1–S7).

The newly sequenced *Mazama chunyi* is consistently placed as a sister taxon to *M. gouazoubira*, whereas the three *M. bricenii* specimens are primarily a sister taxon to *M. rufina*.

The four *P. mephistophiles* specimens always form a clade, which is either a sister taxon to or nested within Odocoileini. Interestingly, they are not placed in a sister position to the mitochondrial genome sequence labelled *P. mephistophiles* from Hassanin et al. (2012). In none of our topologies did *P. mephistophiles* and *P. puda* form a sister taxon relationship, which makes the monophyly of the genus questionable. *M. nemorivaga*, *M. rufina*, *M. bricenii*, *P. puda*, and particularly *P. mephistophiles* occasionally take up positions outside the above proposed clades, thus underpinning their yet unsolved systematics.

Regardless of the controversies debated here and elsewhere regarding Odocoileini molecular systematics, topologies (in the literature and here, Figs. 3 and 4, Figs. S1–S7) show two consistently occurring subclades carrying phylogenetic signal within Odocoileini (e.g., Gilbert, Ropiquet & Hassanin, 2006; Duarte, González & Maldonado, 2008; Hassanin et al., 2012). One subclade consists of *Hippocamelus*, *Blastocerus*, *Ozotoceros*, *M. gouazoubira*, *M. chunyi*, *M. nemorivaga*, and *Pudu puda*. The other subclade consists of *Odocoileus*, *M. americana*, *M. bororo*, *M. nana*, *M. temama*, *M. pandora*, *M. rufina*, and *M. bricenii*. Based on these results we establish two new subtribes Blastocerina and Odocoileina according to the rules of the ICZN (http://www.iczn.org/code). These two subtribes form the tribe Odocoileini and have Rangiferini as sister taxon.

### *Blastocerina subtribus* nova

**Table:** 

Type genus: *Blastocerus* Wagner, 1844
Higher taxa: Odocoileini—Capreolinae—Cervidae

The subtribe Blastocerina consists of the following species: *Blastocerus dichotomus*, *Hippocamelus antisensis*, *Hippocamelus bisulcus*, *Mazama chunyi*, *Mazama gouazoubira*, *Mazama nemorivaga*, *Ozotoceros bezoarticus*, and *Pudu puda* (Fig. 4). Blastocerina refers to the clade originating from the most recent common ancestor of *Blastocerus dichotomus* (Illiger, 1815) and *Pudu puda* Molina, 1782. *Pudu mephistophiles* potentially falls within that clade, but more data are needed for a definite placement of this taxon.

### *Odocoileina subtribus* nova

**Table:** 

Type genus: *Odocoileus* Rafinesque, 1832
Higher taxa: Odocoileini—Capreolinae—Cervidae

The subtribe Odocoileina consists of *Mazama americana*, *Mazama bororo*, *Mazama bricenii*, *Mazama nana*, *Mazama pandora*, *Mazama rufina*, *Mazama temama*, *Odocoileus hemionus*, and *Odocoileus virginianus* (Fig. 4). Odocoileina refers to the clade originating from the most recent common ancestor of *Odocoileus virginianus* (Von Zimmermann, 1778–1783) and *Mazama bricenii* Thomas, 1908.

## Discussion

### Phylogenetic analyses

Our results represent the most complete compilation of molecular data in terms of taxon sample for cervids to date. The thorough sampling enabled us to place the de novo sequenced species in topologies representing overall cervid systematics. We were able to solve some relationships but also discovered previously unknown issues. The data set excludes *Muntiacus gongshanensis*, for which only a very short tRNA sequence is available, and *Axis calamianensis*, *M. montanus*, *M. puhoatensis*, and *M. vaginalis*, for which no molecular data are available.

Our experiments with different matrix sizes, partitioning schemes, and models revealed that the resulting topologies do not dramatically differ from each other. However, we could observe that the resolution decreased with decreasing sequence length. All seven analyses recovered major clades within Cervidae (Table 4 and Fig. 3). These experiments were undertaken to single out strong phylogenetic signal and the significance thereof, which is consistent regardless of the data set sizes and parameter changes. We observed that taxa, which are generally unstable across topologies from different studies (e.g., Pitra et al., 2004; Gilbert, Ropiquet & Hassanin, 2006; Agnarsson & May-Collado, 2008; Hassanin et al., 2012), were the first to lose a supported systematic position with decreasing sequence length (Fig. 3 and Table 4). The partitioning scheme and model choice did not make as much difference as did the matrix size. As expected, partitioning did not necessarily lead to better resolved topologies or significantly better supported clades. However, some differences were observed comparing maximum likelihood with Bayesian inference methods (Figs. 3 and 4).

The topology resulting from re-analysis of the mitochondrial genome sequences (BI-mtG) representing the largest sequence length is fully resolved and has the highest overall support values. The shortest data set (BI-569-unpart), although less well resolved, recovered all higher-level lineages and is in most points congruent with the other topologies based on larger data sets (Table 4 and Fig. 3). These different analyses enabled us to examine the significance of the individual resulting topologies.

### Muntiacus atherodes

The species diversity of Muntiacini is the least covered among cervid subclades in molecular phylogenetic analyses. Muntiacini comprises muntjacs (*Muntiacus*) and the tufted deer (*Elaphodus*), includes the smallest members of Cervinae (40 to 70 cm shoulder height), and inhabits Southeast Asia and Eastern China (Mattioli, 2011). The systematic relationships within Muntiacini in our topologies (Fig. 4) are largely congruent with most recent studies and are the least controversial in molecular cervid systematics (e.g., Pitra et al., 2004; Gilbert, Ropiquet & Hassanin, 2006; Agnarsson & May-Collado, 2008; Hassanin et al., 2012). Here, *M. crinifrons* and *M. feae* are always sister taxa, and when the resolution is sufficiently high, *M. muntjak* is a sister taxon to both of them. In our topologies, *M. putaoensis*, *M. rooseveltorum*, *M. truongsonensis*, and *M. vuquangensis* always form a clade. Most often, with *M. reevesi* is a sister taxon to that clade, but occasionally, *M. reevesi* is sister taxon to all other muntjacs (BI-747-part, BI-569-unpart). Due to the consistent position of *M. muntjak* 2 (AF042718) as sister taxon to *M. truongsonensis*, we suggest re-confirming this sequence.

The monotypic *Elaphodus cephalophus*, which is distributed in southeast China, is always a sister taxon to all muntjacs in both our topologies and previously published trees (Pitra et al., 2004; Gilbert, Ropiquet & Hassanin, 2006; Agnarsson & May-Collado, 2008; Hassanin et al., 2012).

Because of the presumed primitive antler morphology of *M. atherodes* (Groves & Grubb, 1982), its systematic position was hypothesised to be between *Elaphodus cephalophus* and the *Muntiacus*-clade, which is not supported by our results. The newly sequenced holotype specimen of *M. atherodes* is nested within muntjacs, unresolved in a polytomy in most of our topologies. However, some results indicate a potential closer relationship to *M. muntjak* than to any other muntjac. The predominant separate placement from all other *Muntiacus* spp. is an interesting outcome that strengthens the species status of *M. atherodes*.

Several authors assumed the sympatric existence of a second muntjac species on Borneo that was separate from *M. muntjak* (Kohlbrugge, 1895; Lyon Jr, 1911; Van Bemmel, 1952; Hill, 1960) before Groves & Grubb (1982) eventually established *M. atherodes* based on a skin and the holotype skull sampled for the present study. The endemic *M. atherodes* differs from *M. muntjak* in colouration and has smaller, simpler antlers, and the latter has a much wider distribution across Southeast Asia and Southern China (Groves & Grubb, 1982).

Though unsupported, the potential close systematic relationship of *M. atherodes* and *M. muntjak* would be logical based on the endemic occurrence of *M. atherodes* on Borneo. *M. atherodes* and *M. muntjak* could have diverged from a common ancestor on Borneo via sympatric speciation and with a later invasion of *M. muntjak* to the mainland.

Alternatively, *M. muntjak* could have invaded Borneo during the sea level fluctuations in the Plio-Pleistocene (Voris, 2000; Meijaard, 2003; Woodruff, 2003; Meijaard & Groves, 2004; Bibi & Métais, 2016), resulting in the allopatric speciation of *M. atherodes* and its isolation from the mainland populations during the end-Pleistocene sea level rise.

The high sea levels in the early Pliocene split the Thai-Malayan Peninsula into two landmasses, which separated Indochinese from Sundaic faunas (Woodruff, 2003). This most likely had a large influence on the evolution of Southeast Asian cervids and probably occurred again later during the Pliocene (Meijaard & Groves, 2004). Sea level changes in the Malay Archipelago were important for faunal dispersals. Low sea levels allowed species to spread to landmasses, which would become islands with rising sea levels, resulting in isolation of populations.

Detailed descriptions and maps for sea level changes of Southeast Asia can be viewed in Voris (2000) and Meijaard (2003).

### Rusa marianna

In the literature, there is a broad consensus about the systematic relationships within Cervini. However, the taxonomy of *Cervus* s. l. is indeed complicated (Randi et al., 2001). The controversy primarily concerns delimitations of genera and/or subgenera. *Rusa*, *Rucervus*, *Przewalskium* (= *Cervus*) *albirostris*, and *Cervus* are occasionally treated as subgenera of the genus *Cervus*, whereas *Axis*, *Elaphurus*, and *Dama* are normally treated as separate genera (Groves & Grubb, 1987; Randi et al., 2001). Here, we refer to *Rucervus* and *Rusa* as individual genera and refer to *Przewalskium albirostris* as *Cervus albirostris*.

The four species of *Rusa*, *R. alfredi*, *R. marianna*, *R. timorensis*, *R. unicolor*, inhabit India, Indochina and the Malay-Archipelago (Grubb & Groves, 1983; Mattioli, 2011). *R. unicolor* is the largest oriental deer and has a highly fragmentary distribution from southern Nepal, India and Sri Lanka along the southern Himalayas through to mainland Southeast Asia and many of the Greater Sunda islands (Timmins et al., 2008; Leslie, 2011). *R. timorensis* is endemic to the Indonesian islands Bali and Java (Hedges et al., 2008). *Rusa alfredi* is one of the rarest deer species according to the IUCN Red List (IUCN, 2015) and is endemic to Panay and Negros (Western Visayan Islands, Central Philippines) (Oliver et al., 2008). In contrast, *Rusa marianna* is more widely distributed across most of the Philippine Islands, with the exceptions of the Negros-Panay, Sulu and Palawan Faunal Region, the Babuyan/Batanes groups, and other isolated islets (MacKinnon, Ong & Gonzales, 2008).

The four newly sequenced individuals of *Rusa marianna* are positioned to be closely related to each other in a distinct clade. Two of the individuals are in a polytomy with the other Philippine species, *Rusa alfredi*, and two form a clade, which is a sister taxon to the polytomy (Fig. 4). Our topology supports the hypothesis that the two Philippine *Rusa* species are closely related and are sister taxon to *R. timorensis* and *R. unicolor*.

Investigations by Grubb & Groves (1983) showed that interpreted relationships within *Rusa* are controversial. *Rusa timorensis* and *R. unicolor* are sister taxa supported in all our topologies (Fig. 4, Figs. S1–S7), and this clade is in a polytomy with the *Cervus*-clade (including *C. albirostris*) and the *R. alfredi*-*R. marianna*-clade. A close relationship between *Rusa* and *C. albirostris* was already suggested by Flerov (1952) based on morphological evidence and a supposed divergence of *C. albirostris* from *Rusa* in the Late Pliocene.

The evident phenotypic separation of spotted (*R. alfredi*) and non-spotted (*R. marianna*) *Rusa* deer on the Philippines suggests two invasion events (Grubb & Groves, 1983), but the missing molecular data for *R. marianna* have prohibited further explanations. Grubb & Groves (1983) suggested a Southeast Asian mainland common ancestor from which a peripheral population diverged by evolving into *R. timorensis*. Later, a population of those colonised the Philippines twice at early and later stages in diversification, evolving into *R. alfredi* and *R. marianna*. *R. unicolor* evolved there but failed a third colonisation on additional Philippine Islands and dispersed northwards to the mainland. Meijaard & Groves (2004) pointed to the likely high impact of Plio-Peistocene sea level fluctuations on Southeast Asian cervid dispersal and speciation.

However, the suggested speciation of *R. marianna* and *R. alfredi* is not clearly evident from our topologies, where *R. alfredi* appears to be a subgroup of *R. marianna* rather than a sister taxon. More data are needed to unambiguously solve their relationships.

### Odocoileini

Odocoileini represents the most controversial subclade of extant cervids. They consistently split into two subclades in both our current results and previously published phylogenetic trees. For these two subclades we established the new subtribes Blastocerina and Odocoileina (see above). However, within each of these subclades, systematic relationships are not yet solved. The recent divergence of modern neotropical Odocoileini from extinct Eurasian Capreolinae and related insufficient genomic diversity available to solve systematic relationships could be the reason (Vislobokova, 1980). All genera except for *Odocoileus* are endemic to South America, and their ancestors reached the continent via the Panamanian Isthmus in the Pliocene (5–2.5 million years ago) (Webb, 2000; Gilbert, Ropiquet & Hassanin, 2006). The first fossil appearances are known from no longer than approximately 2.4 million years ago (Webb, 2000). The consistent split of Blastocerina and Odocoileina potentially represents an asynchronous dispersal history via two invasion events.

Furthermore, our study revealed dubious relationships between available *Hippocamelus* sequences. All of our topologies (Fig. 4, Figs. S2–S7) show that two *H. antisensis* sequences (*H. antisensis* 1; JN632646, NC_020711 (Hassanin et al., 2012)) are a sister taxon to *H. bisulcus*. However, the other two sequences (*H. antisensis* 2; DQ379307 (Gilbert, Ropiquet & Hassanin, 2006) and GU190862 (Fuentes-Hurtado et al., 2011)), are a sister taxon to *Ozotoceros* in all of our topologies (Fig. 4, Figs. S2–S7). This is a critical issue, although its resolution is beyond the scope of this study; however, we found it important to point to this drawback in the base data and suggest re-confirmation of all four sequences.

Systematics of the two dwarfed genera, *Mazama* and *Pudu*, whose small body size and simplified antlers are interpreted as secondary adaptations to dense vegetation (Geist, 1998; Mattioli, 2011), are particularly uncertain. Their habitat use and their decline in individual numbers makes it increasingly difficult to obtain enough data to resolve systematic issues from some of the species (see below).

### Pudu

Pudus are the smallest living deer (25 to 40 cm shoulder height) and the smallest New World hoofed mammals (Hershkovitz, 1982; Mattioli, 2011). It is difficult to distinguish both *pudu* species from sympatric small deer species (*Mazama*) based only on the phenotype, without direct comparison (Hershkovitz, 1982; Jiménez, 2011). *Pudu* and *Mazama* likely represent divergent lineages of small odocoilein deer (Hershkovitz, 1982). Although the origin of pudus is unknown, Hershkovitz (1982) stated that *P. mephistophiles* has more primitive phenotypical features than *P. puda*.

*Pudu* was assumed to be polyphyletic (Hassanin et al., 2012). Whereas *P. puda* has been well-sampled and studied, information for *P. mephistophiles* is scarce. In all of our topologies (Fig. 4, Figs. S1–S7), the four newly sequenced specimens of *Pudu mephistophiles*, including the holotype, form a well-supported clade. However, the position of that clade is variable. In four topologies (BI-1140-unpart, BI-747-part, BI-747-unpart, BI-569-unpart; Fig. 4, Figs. S2, S5, S6 and S7), the clade is a sister taxon to all other Odocoileini and Rangiferini; in one topology (ML-1140; Fig. S4), it is a sister taxon to all Blastocerina with poor support; and in one topology (BI-1140-part; Fig. 3C, Fig. S3), it is placed in an unresolved position with other Odocoileini clades and Rangiferini. The placement of the individual *Pudu mephistophiles* specimen published prior to our study in Hassanin et al. (2012) (JN632691) is not close to the *P. mephistophiles*-clade in our topologies. Instead, it is placed as a sister taxon to *Mazama rufina* (Fig. 4, Figs. S1–S7) and confirms Hassanin et al.’s (2012) suspicion that it might in fact be a misidentified *Mazama rufina* and is neglected for further interpretation. The holotype specimen included in the four new *P. mephistophiles* samples substantiates that suspicion. In all but one topology (BI-569-unpart), *P. puda* is a sister taxon to all other Blastocerina, which is congruent with Hassanin et al. (2012) and Agnarsson & May-Collado (2008). In Duarte, González & Maldonado (2008), however, its position was unresolved. The placement of *P. mephistophiles* separate from its congeneric *P. puda* in most topologies suggests polyphyly of the genus.

### Mazama

The genus *Mazama* comprises several species of small- to medium-sized deer (40 to 80 cm shoulder height) (Hershkovitz, 1959; Hershkovitz, 1982; Mattioli, 2011). The current distribution of *Mazama* ranges from Southern Mexico to Argentina (IUCN Red List, Mattioli, 2011; González et al., 2009).

Since the first description of *Mazama pita* Rafinesque, 1817 (= *Moschus americanus* Erxleben, 1777), the genus has been subject to taxonomic controversies. Allen (1915) recognised 18 species of *Mazama*; Cabrera (1960) reduced these to four species, i.e., *M. chunyi*, *M. gouazoubira*, *M. nana*, and *M. rufina*. Czernay (1987) established two more species, *M. americana* and *M. bricenii*, whereas Groves & Grubb (1987) considered *M. temama* a possible separate species based on cytogenetic differences. Medellín, Gardner & Marelo Aranda (1998) revised *M. pandora* as a separate species based on differences in the skulls and skins. Rossi (2000) established *M. nemorivaga* as a fourth sympatric species in Brazil (together with *M. americana*, *M. nana*, *M. gouazoubira*). Duarte (1992) described *M. bororo* based on karyotype differences, which adds up to ten *Mazama* species being widely accepted today (IUCN Red List, Mattioli, 2011; González et al., 2009). More recently, Abril & Duarte (2008) recognised only eight species (*M. americana*, *M. bororo*, *M. chunyi*, *M. gouazoubira*, *M. nana*, *M. nemorivaga*, *M. pandora*, and *M. rufina*), whereas Groves & Grubb (2011) listed 24 different species of *Mazama*. Most of the species share phenotypic similarities, which makes their discrimination almost impossible; however, there are differences in overall body size, coat colour, and/or karyotype (González et al., 2009).

Recently, polyphyly of *Mazama* was observed (Duarte, González & Maldonado, 2008; Hassanin et al., 2012). Within Odocoileina, Duarte, González & Maldonado (2008) found a separation of the genus into a mixed *Mazama americana*-clade that included *M. bororo* and *M. nana*. *M. americana* appeared polyphyletic because there was an additional clade consisting exclusively of *M. americana* as a sister taxon to *Odocoileus* and the mixed *M. americana*-clade (Duarte, González & Maldonado, 2008). Hassanin et al. (2012) found *M. americana* to be monophyletic and a sister taxon to *Odocoileus*. *M. rufina* is a sister taxon to the *Mazama*-*Odocoileus*-clade (Hassanin et al., 2012).

Within Blastocerina there were two clades: a *Mazama gouazoubira*-clade and a *M. nemorivaga*-clade. Their position varies from study to study (Agnarsson & May-Collado, 2008; Duarte, González & Maldonado, 2008; Hassanin et al., 2012).

In our topologies, within Odocoileina, the mixed *Mazama americana*-clade that includes the sequences indicated as *M. americana* 1–3 is supported (Fig. 4) and has the most stable position, forming the sister taxon to the *Odocoileus*-clade. The pure *M. americana*-clade found by Duarte, González & Maldonado (2008) is represented in our topology by the sequences indicated as *M. americana* 4 and *M. americana* 5.

*M. rufina* is nested within Odocoileina and is a sister taxon to the *Mazama*-*Odocoileus*-clade (BI-1140-unpart, BI-1140-part, ML-1140; Figs. 3 and 4, Figs. S2, S3 and S4) or is placed in resolved or unresolved positions outside Odocoileina but within Odocoileini (BI-747-unpart, BI-747-part, BI-569-unpart; Fig. 3, Figs. S5, S6 and S7).

*M. gouazoubira* is either a sister taxon to both *Hippocamelus* species (BI-747-unpart, BI-569-unpart; Fig. 4, Figs. S5 and S7), or *Blastocerus* is placed between *Hippocamelus* and *M. gouazoubira*. *M. gouazoubira* itself is polyphyletic in our topologies (Fig. 4), and a re-confirmation of the *M. gouzoubira* 2 sequence (DQ379308 (Gilbert, Ropiquet & Hassanin, 2006)) is suggested.

Finally, the *M. nemorivaga*-clade is mostly nested within Blastocerina or is placed unresolved within Odocoileini (BI-747-part, BI-569-unpart).

In our study, *M. temama* and *M. pandora* were included in a species-rich phylogenetic analysis of cervids with palaearctic and neotropical species for the first time. Similarly to recent results of Escobedo-Morales et al., 2016, our results show that *M. temama* is always within Odocoileina as a sister taxon to the mixed *M. americana*-clade. In Escobedo-Morales et al., 2016 and in our topologies, *M. pandora* is consistently placed within Odocoileina as a sister taxon to *Odocoileus*.

This also indicates a critical issue concerning the dispersal history of South American cervids. The placement of the *M. americana*-splits in Fig. 4 can be alternatively interpreted as a paraphyletic *M. americana*-clade, within which all other species are nested, i.e., *Odocoileus* sp., *M. pandora*, *M. temama*, *M. nana*, and *M. bororo*. However, the placement of *M. temama* disrupts the continuous genealogy of *M. americana*. Together with the clade consisting of *M. rufina* and *M. bricenii* (see below), Odocoileina is basically a *Mazama*-clade, within which *Odocoileus* diverged and *Mazama* diversified into several species. This scenario would strongly question the long-held assumption that *Odocoileus* was the first cervid to immigrate to South America and diversify into the extant South American species (Anderson & Wallmo, 1984; Smith, 1991; Geist, 1998) (see also Escobedo-Morales et al., 2016).

Our results from sequencing *M. chunyi* show a sister taxon relationship with *M. gouazoubira* within Blastocerina in all our topologies (Fig. 4). The newly sequenced *Mazama bricenii* specimens are always placed in a sister taxon position to *M. rufina* in our topologies but exist as a monophyletic group in only one topology (BI-569-unpart; Fig. 3, Fig. S7).

In two topologies, the specimen BMNH 1908.6.24.5 is placed isolated from the other two specimens (BMNH 1913.4.24.3, BMNH 1934.9.10.228), which remain sister taxa to *M. rufina*. Specifically, in one topology, BMNH 1908.6.24.5 is in an unresolved position within Odocoileina (BI-747-part; Fig. 3, Fig. S6) and is positioned as a sister taxon to *M. chunyi* in the other topology (BI-1140-part; Fig. 3, Fig. S3).

Mattioli (2011) listed *M. bricenii* and *M. chunyi* as subspecies of *M. rufina*. The *Mazama bricenii* specimen BMNH 1934.9.10.228 was originally assigned to *M. rufina*. Additionally, its sampling locality in Ecuador is outside the assumed current distribution of *M. bricenii* (Fig. 2 and Table 2) and thus makes the revised affiliation to *M. bricenii* questionable. *M. bricenii* is scarcely distributed in Northeast Colombia and West Venezuela, whereas *M. rufina* is distributed along the Andes from central Colombia to Ecuador and North Peru (Weber & González, 2003; Lizcano, Álvarez & Delgado-V, 2010). This distribution is intermediate between the distribution of *M. bricenii* and *M. chunyi*. The latter is certainly known from South Peru and North Bolivia based on isolated museum specimen localities and rare sightings in the wild. Equally scarce is information on the biology and ecology of these species (Rumiz & Pardo, 2010). The results of the most recent study on systematic relationships of *M. bricenii* based on *Cytb* confirm our results and suggest that *M. bricenii* is a junior synonym of *M. rufina* (Gutiérrez et al., 2015).

Despite the extensive taxonomic and phylogenetic interest in the genus *Mazama* due to unsolved questions, the taxon remains enigmatic (e.g., Duarte & Merino, 1997; Medellín, Gardner & Marelo Aranda, 1998; Duarte & Jorge, 2003; Weber & González, 2003; Duarte, González & Maldonado, 2008; González et al., 2009). In particular, the high intraspecific variability in *M. americana* and *M. gouazoubira* stimulated additional taxonomic and genetic research on the genus (see Weber & González, 2003). The systematics of *M. americana* is particularly problematic because even the species appears polyphyletic with possible cryptic species (Duarte, González & Maldonado, 2008; Abril et al., 2010). Abril et al. (2010) showed that *M. americana* exhibits an extensive karyotype variation and found two distinct clades within *M. americana* sampled across Brazil. They also found that one clade is more closely related to *M. bororo* and *M. nana*, presumably corresponding to *M. americana* 1–3 in our topology, than to the second (pure) clade of *M. americana* (Fig. 4). Additionally, the genetic distance between the *M. americana*-clades was higher than that between *M. nana* and *M. bororo*. This suggests two separation events in the two lineages of *M. americana* (Abril et al., 2010). There is the potential that even more species are hidden in both the *M. americana*-complex and the *M. gouazoubira*-complex (Weber & González, 2003). Cytogenetics seems to be the most reliable technique for distinguishing between sympatric species (Vogliotti & Duarte, 2009). Much more data and thorough research on *Mazama* are needed to shed additional light on their complex systematic relationships.

## Conclusion

The taxonomically most extensive molecular phylogenetic data set for cervids compiled to date enabled us to undertake phylogenetic analyses to answer and test the initial questions and hypotheses: (1) *Mazama bricenii* is closely related to *M. rufina* and is more closely related to the *M. americana*-clade than to the *M. gouazoubira*-clade. However, from our topology, we infer that *M. rufina* is a subclade of *M. bricenii*. It cannot be excluded that these two taxa may represent the same species with *M. rufina* as the senior synonym. *Mazama chunyi* forms a sister taxon relationship with *M. gouazoubira* and can thus be assigned to the *M. gouazoubira*-clade. The discovery of a fifth clade (*M. pandora*) shows that the polyphyly and systematic relationships within *Mazama* are even more complex than previously thought and remain a challenge to address in future research. (2) *Muntiacus atherodes* is supported to be a valid species distinct from other *Muntiacus* spp. However, its systematic position cannot be resolved with certainty, but the maximum likelihood analysis indicates that it might be more closely related to the sympatric *M. muntjak* than to any other muntjac. (3) The Philippine rusine deer *R. marianna* and *R. alfredi* form a monophyletic clade and are sister taxon to a clade containing the other rusine deer, *R. timorensis* and *R. unicolor* and to the *Cervus*-clade. Our results indicate that *R. alfredi* forms a subclade of *R. marianna* rather than its sister taxon. (4) The genus *Pudu* appears to be polyphyletic, with *P. puda* nested within the Blastocerina and *P. mephistophiles*, thereby forming a monophyletic group in a yet-unresolved position.

Based on our topologies and previous work, we established here the new subtribes Blastocerina and Odocoileina, which form Odocoileini. A revision of the current taxonomy based on comparison of phenotypic and genotypic traits is desirable for future research on cervid systematics.

##  Supplemental Information

10.7717/peerj.2307/supp-1 Supplemental Information 1 Supplementary Information This file contains the figures of the resulting topologies from all undertaken phylogenetic analyses in this study.

10.7717/peerj.2307/supp-2 Supplemental Information 2 Matrix This file contains the full 1,140 base pair long cytochrome b data matrix.

## Additional Information and Declarations

### DNA Deposition

The following information was supplied regarding the deposition of DNA sequences: European Nucleotide Archive: LT546647 –LT546659.

### Data Availability

The following information was supplied regarding data availability: All alignments and analyses information are deposited at Open Data LMU doi: 10.5282/ubm/data.96.
