# Supplementary material for: Systematic relationships of five newly sequenced cervid species
Source: PeerJ. 2016 Aug 4;4:e2307. doi: 10.7717/peerj.2307 (PMC4991894; doi:10.7717/peerj.2307)
Supplement: Supplemental Information 1 — This file contains the figures of the resulting topologies from all undertaken phylogenetic analyses in this study. [file peerj-04-2307-s001.docx]

**Supplementary Information**

**Strengths, weaknesses, opportunities, and threats in current cervid systematics as revealed by newly retrieved ancient DNA**

Nicola S. Heckeberg^1,2,3^, Dirk Erpenbeck^1,4^, Gert Wörheide^1,2,4^, Gertrud E. Rössner^2,1,4^

^1^Department of Earth and Environmental Sciences, Palaeontology & Geobiology, Ludwig-Maximilians-Universität München, Munich, Germany

^2^SNSB – Bavarian State Collection for Palaeontology and Geology, Munich, Germany

^3^Department of Zoology, University of Cambridge, Cambridge, UK

^4^GeoBio-Center^LMU^, Munich, Germany

**Figure S1.** BI-mtG. Bayesian topology of the re-analysis of the complete mitochondrial genome data set published by Hassanin et al. (2012) using their partitioning and model scheme.

**Figure S2.** BI-1140-unpartitioned. Bayesian Inference topology based on the unpartitioned data set of the complete cytochrome b sequence (1140 bp) using GTR+Γ. This is the detailed view of the tree in the main text figure 4.

**Figure S3.** BI-1140-partitioned. Bayesian topology based on the partitioned data set of the complete cytochrome b sequence (1140 bp) using SYM, HKY, and GTR for the 1^st^, 2^nd^, 3^rd^ codon position, all with the Γ-distribution.

**Figure S4.** ML-1140. Maximum Likelihood topology based on the partitioned data set (per codon position) of the complete cytochrome b sequence (1140 bp) using GTR+Γ.

**Figure S5.** BI-747-unpartitioned. Bayesian topology of the unpartitioned data set of 747 bp long cytochrome b sequence using GTR for all three codon position, all with the Γ-distribution.

**Figure S6.** BI-747-partitioned. Bayesian topology of the partitioned data set of 747 bp long cytochrome b sequence using SYM, HKY, and GTR for the 1^st^, 2^nd^, 3^rd^ codon position, all with the Γ-distribution.

**Figure S7.** BI-569-unpartitioned. Bayesian topology of the unpartitioned data set of the cytochrome b sequence reduced to 569 bp using GTR+Γ.

**References**

Hassanin A, Delsuc F, Ropiquet A, Hammer C, Jansen van Vuuren B, Matthee C, Ruiz-

Garcia M, Catzeflis F, Areskoug V, Thanh Nguyen T, Couloux A. 2012. Pattern and timing of diversification of Cetartiodactyla (Mammalia, Laurasiatheria), as revealed by a comprehensive analysis of mitochondrial genomes. *Comptes Rendus Biologies*, 335(1):32–50.
